# Calcium and Vitamin D Supplementation and Their Association with Kidney Stone Disease: A Narrative Review

**DOI:** 10.3390/nu13124363

**Published:** 2021-12-04

**Authors:** Matteo Bargagli, Pietro Manuel Ferraro, Matteo Vittori, Gianmarco Lombardi, Giovanni Gambaro, Bhaskar Somani

**Affiliations:** 1 U.O.C. Nefrologia, Dipartimento di Scienze Mediche e Chirurgiche, Fondazione Policlinico Universitario A. Gemelli IRCCS, 00168 Roma, Italy; matteo.bargagli@unicatt.it (M.B.); pietromanuel.ferraro@unicatt.it (P.M.F.); 2 Dipartimento Universitario di Medicina e Chirurgia Traslazionale, Università Cattolica del Sacro Cuore, 00168 Roma, Italy; 3 Department of Urology, San Carlo di Nancy Hospital, 00165 Rome, Italy; mvittori@gvmnet.it; 4 U.O.C. Nefrologia, Azienda Ospedaliera Universitaria Integrata di Verona, 37126 Verona, Italy; gianmarcolombardi87@gmail.com (G.L.); giovanni.gambaro@univr.it (G.G.); 5 Department of Urology, University Hospital Southampton NHS Trust Southampton, Southampton SO16 6YD, UK

**Keywords:** kidney stones, calcium, vitamin D

## Abstract

Kidney stone disease is a multifactorial condition influenced by both genetic predisposition and environmental factors such as lifestyle and dietary habits. Although different monogenic polymorphisms have been proposed as playing a causal role for calcium nephrolithiasis, the prevalence of these mutations in the general population and their complete pathogenetic pathway is yet to be determined. General dietary advice for kidney stone formers includes elevated fluid intake, dietary restriction of sodium and animal proteins, avoidance of a low calcium diet, maintenance of a normal body mass index, and elevated intake of vegetables and fibers. Thus, balanced calcium consumption protects against the risk for kidney stones by reducing intestinal oxalate availability and its urinary excretion. However, calcium supplementation given between meals might increase urinary calcium excretion without the beneficial effect on oxalate. In kidney stone formers, circulating active vitamin D has been found to be increased, whereas higher plasma 25-hydroxycholecalciferol seems to be present only in hypercalciuric patients. The association between nutritional vitamin D supplements and the risk for stone formation is currently not completely understood. However, taken together, available evidence might suggest that vitamin D administration worsens the risk for stone formation in patients predisposed to hypercalciuria. In this review, we analyzed and discussed available literature on the effect of calcium and vitamin D supplementation on the risk for kidney stone formation.

## 1. Introduction

Kidney stone disease is characterized by high prevalence and progressively increasing incidence in both genders worldwide [1,2,3]. This condition is also frequently associated with different comorbidities, including cardiovascular risk factors and vascular calcifications [4,5], higher body mass index [6,7], diabetes [8], metabolic syndrome [9,10], and higher risk for chronic kidney disease development [11,12]. The most common stone components are calcium-based, either in association with oxalate or, less frequently, with phosphate, representing up to 75% of all stone phenotypes, whereas the prevalence of uric acid stones is only around 10% [13]. Furthermore, mixed stones also exist, including mixed calcium oxalate and uric acid stones. The pathophysiology of calcium nephrolithiasis starts from nucleation, followed by crystal aggregation and growth [14]. Among other factors, low urine volume, reduced urinary magnesium and citrate concentrations, unduly acidic or alkaline urine pH, and increased urinary excretion of calcium, oxalate, and uric acid also contribute to stone formation [15,16,17].

Kidney stone disease possesses a multifactorial origin, including both genetic predisposition and environmental risk factors [18,19,20]. Dietary habits belong to the latter group, being one of the most common risk factors for kidney stones and especially for uric acid and calcium phenotypes. In fact, general nutritional advice for calcium stone formers includes increased fluid intake, balanced calcium intake, reduced dietary intake of sodium and animal proteins, maintenance of a healthy body mass index, and increased intake of vegetables and fibers [21].

While the available literature on calcium supplementation and dietary calcium intake agrees on the harmful effect of low calcium diets on the risk of stone formation, data on vitamin D consumption are less consistent [22].

Here, we review the available evidence of calcium and vitamin D supplementation on the risk for kidney stone formation.

## 2. Materials and Methods

In this narrative review, we searched all the available literature regarding the association between dietary calcium intake and vitamin D supplementation with kidney stone disease. We selected articles from several online databases, including: PubMed, the Cochrane Library, and Web of Science. Articles included in the present study were obtained using the following definitions: (“dietary calcium” OR “calcium supplementation” OR “calcium intake” OR “calcium consumption” OR “25(OH)D” OR “1,25(OH)2D” OR “25-hydroxyvitamin D” OR “1,25-dihydroxyvitamin D” OR “25-hydroxycholecalciferol” OR “1,25-dihydroxycholecalciferol” OR “vitamin D” OR “cholecalciferol” OR “calcitriol” OR “calcifediol” OR “ “calcium uptake” OR “calcium absorption”) AND (“nephrolithiasis” OR “kidney stone*” OR “kidney calculi” OR “urolithiasis” OR “urinary calcium excretion”). No restriction was made based on the publication language, type of article, or year of publication. A total of 115 references were included in this review.

## 3. Physiology of Bone and Mineral Metabolism

In physiological conditions, bone turnover is finely tuned by bone cells, including osteoclasts, osteoblasts, and osteocytes [23,24].

Bone tissue is the main storage for total body calcium [23], and its homeostasis is controlled by specific hormones (PTH, 1,25-dihydroxycholecalciferol, and calcitonin) that exert their effects on three different systems: kidney, bone, and gastrointestinal tract [25].

Normally, PTH and 1,25-dihydroxycholecalciferol secretions are induced by reduced circulating serum calcium, consequently increasing both its renal retention and intestinal absorption. On the contrary, PTH secretion is inhibited by hypercalcemia [26].

Intestinal calcium uptake is both transcellular and paracellular, and the former is increased by calcitriol activation. At the bowel level, calcium uptake is managed by two distinct mechanisms: a passive, paracellular transport, taking place in all intestine segments, and an active, transcellular process, mainly located in the duodenum and jejunum. The latter involves intestinal brush border, calbindin-mediated intracellular diffusion, and absorption. In case of low calcium intake, the majority of calcium uptake is transcellular. On the contrary, when dietary calcium consumption is very high, the calbindin pathway is downregulated [26,27,28].

The kidneys also have several mechanisms for regulating serum calcium homeostasis, acting on urinary calcium excretion. In physiological conditions, filtered calcium is reabsorbed both transcellularly and paracellularly. The former occurs mainly in the distal convoluted tubule and is regulated by the activity of PTH and active vitamin D [29]. The latter takes place in the proximal tubule and thick ascending limb, in accordance with active sodium reabsorption, and it is controlled by the calcium-sensing receptor, which is capable of inhibiting paracellular calcium transport by upregulating claudin-14, blocking tubular permeability to divalent cations [30,31].

## 4. Evidence of Bone and Mineral Metabolism Derangements in Patients with Kidney Stone Disease

The most frequent stone phenotypes are calcium-based, mainly in the form of calcium oxalate and calcium phosphate, either pure or mixed with another constituent, such as uric acid [32]. The risk for calcium phosphate kidney stones is caused by a high urinary pH that, in turn, increases calcium phosphate supersaturation [33]. On the contrary, the calcium oxalate phenotype yields a completely different etiology, with a higher risk in the case of higher urinary calcium and/or oxalate excretion [32]. However, additional risk factors for stone formation have to be considered, including low urine volume, low urine citrate, increased urinary uric acid excretion, and high dietary acid load from foods [17,34,35,36]. Thus, high urinary calcium excretion is one of the major risk factors for calcium kidney stones [37], but a true cut-off for the likelihood of stone formation based on urinary calcium excretion does not exist, and higher risk can already be found when calciuria exceeds 150–200 mg/24 h [38,39].

Nevertheless, patients with high urinary calcium excretion were historically classified based on their alleged etiology in renal calcium leak as either idiopathic, secondary to bone resorption, as in conditions such as primary hyperparathyroidism; or absorptive, due to increased intestinal calcium uptake [40,41]. Diet is also fundamental in the determination of urinary calcium excretion, and elevated sodium consumption decreases renal calcium resorption, increasing the urinary calcium concentration. A Western diet typically marked by high animal protein intake was also shown to be associated with raised urinary calcium excretion, in part due to elevated dietary acid load but also due to additional mechanisms that are not yet completely characterized [39,42,43].

Thus, the gut has a strong influence on urinary solutes excretion. The linkage between intestinal calcium absorption and its urinary excretion has been known for more than 50 years [44,45]. In fact, calcium uptake from the gut takes place both in the transcellular and paracellular space. The former depends on luminal calcium concentration, whereas the latter is increased in the case of active vitamin D stimulation [22,46].

At the bowel level, when the vitamin D receptor (VDR) located on the enterocyte’s nucleus binds calcitriol, transcellular calcium transport increases through the activity of the TRPV6 transporter (gatekeeper transient receptor potential vanilloid 6) [47].

However, the role of circulating calcitriol and 25-hydroxyvitamin D concentrations in the genesis of kidney stone disease is not yet completely clarified [48,49,50,51,52]. A higher 1,25-dihydroxycholecalciferol concentration has been found in calcium stone formers, and a direct association between calcitriol levels and the risk for incident symptomatic nephrolithiasis was also present for subjects without previous stone events [53,54,55,56]. In addition, the administration of ketoconazole in hypercalciuric kidney stone formers, defined as affected by absorptive hypercalciuria, was only capable of reducing urinary calcium excretion in a portion of the patients. Since ketoconazole decreases calcitriol synthesis, it might be supposed that the increase in calcium uptake is caused by several mechanisms, only a portion of which are vitamin D-dependent [57].

The vitamin D receptor can be found in several tissues, including bones, kidneys, the immune system, fat tissues, and parathyroid cells. When the parathyroid VDR is activated from its ligand 1,25-dihydroxycholecalciferol, PTH production is decreased, reducing its effect on urinary calcium reabsorption in the distal tubule, consequently increasing urinary calcium excretion [58,59]. This effect may be found in specific conditions characterized by uncontrolled calcitriol production and activation, such as sarcoidosis and primary hyperparathyroidism. On the contrary, in the case of severe vitamin D deficiency, there is a compensatory increase in PTH secretion for maintaining stable serum calcium concentration by mobilizing calcium from the skeleton and reabsorbing it from the kidneys [60,61]. This defines secondary hyperparathyroidism due to vitamin D deficiency, and it is diagnosed by reduced urinary calcium excretion in the case of normal–low serum calcium [62].

These findings may lead to the hypothesis that different pathogenetic mechanisms are involved other than isolated calcitriol-induced hypercalciuria in patients affected by calcium oxalate stones. In fact, low serum phosphate concentration was also proposed as a hypothetical cause of calcium nephrolithiasis [63]. In the case of reduced serum phosphate, the negative feedback exerted by fibroblast growth factor-23 on calcitriol is blunted [64]. This may lead to higher circulating active vitamin D, increasing intestinal calcium absorption and leading to higher urinary calcium excretion. In support of this theory, mutations of the SLC34A1 gene, encoding for the proximal tubular phosphate transporter NPT2a, were linked to vitamin D-dependent hypercalciuria [65,66]. Another monogenic cause of hypercalciuric nephrolithiasis involves the CYP24A1 gene, encoding for 1,25(OH)2D-24-hydroxylase, the enzyme involved in calcitriol degradation [67,68,69]. Its mutations jeopardize the activity of 24-hydroxilase and induce elevated serum calcitriol concentration that in turn causes hypercalcemia, hypercalciuria, and suppressed PTH secretion [70,71,72]. The clinical spectrum of the conditions induced by homozygous mutations of the CYP24A1 gene spans from idiopathic infantile hypercalcemia in children to recurrent nephrolithiasis and/or nephrocalcinosis with hypercalciuria in adulthood [73,74,75].

However, heterozygous mutations of this recessive gene have also been observed in hypercalciuric stone formers. More recently, a comparison between 30 healthy subjects, hypercalciuric and normocalciuric stone formers, found an increased 1,25-dihydroxycholecalciferol/1,25(OH)2D-24-hydroxylase ratio in both hypercalciuric and normocalciuric stone formers compared to healthy individuals, possibly suggesting that 1,25-dihydroxycholecalciferol degradation is blunted in those patients [76,77].

This evidence may indicate a causative role of these polymorphisms in the epidemiological association of higher calcitriol concentration and stone disease [56].

This hypothesis is supported by the demonstration that calcium kidney stone formers have lower 24,25-hydroxyvitamin D/25-hydroxyvitamin D ratios compared to healthy individuals [78]. This ratio was indeed recently confirmed as an accurate diagnostic tool for detecting CYP24A1 mutations [79].

As described above, not all calcium stone formers have high serum calcitriol concentrations, and a connection to the vitamin D receptor’s increased activity was suggested.

In fact, a rat model of augmented VDR activity in bone, renal, and intestinal tissues was found to be characterized by increased intestinal calcium uptake, bone resorption, and reduced renal tubular calcium reabsorption, causing extremely elevated urinary calcium concentrations [80,81,82,83,84]. Calcitriol supplementation in these rats further increased both intestinal calcium absorption and bone mobilization [85].

Different VDR polymorphisms were actually found to be associated with kidney stones in human patients, but the pathogenic role of this association is yet to be determined [86]. Other authors found no increased expression of VDR in hypercalciuric patients [87].

The high dietary protein consumption was believed to cause an increase in renal mass secondary to glomerular hyperfiltration and a consequent hyper-activation of 25-hydroxycholecalciferol, ultimately leading to hypercalciuria and recurrent kidney stones [88].

## 5. Evidence on the Role of Vitamin D and Calcium Supplementation in Patients with Kidney Stone Disease

### 5.1. Dietary Calcium Intake and Supplementation

Dietary calcium intake is fundamental for body homeostasis, and several systems are influenced by the amount of ingested calcium, including bone health and growth, muscle function, kidney function, glycolysis and gluconeogenesis, intestinal function, and the performance of the parathyroid glands [89]. As mentioned above, in the case of idiopathic hypercalciuria, urinary calcium resorption is defective, possibly causing a negative calcium balance and an increased risk for nephrolithiasis [90].

If extensively supplemented, dietary calcium was shown to raise urinary calcium by up to 20% in patients already affected by hypercalciuria [91]. Therefore, one might presume that reducing dietary calcium will improve hypercalciuria and reduce the risk of crystal formation. Calcium consumption, if not artificially supplemented, does not exceed 1.2 g/day (Table 1). In addition, conditions such as hyperparathyroidism or osteoporosis are associated with a negative calcium balance, increasing urinary calcium excretion regardless of dietary intake [92,93,94]. Furthermore, the connection between dietary calcium and oxalate excretion is well established: calcium works as a chelator for both phosphate and oxalate in the gut; thus, a low calcium diet will increase the amount of intestinal free-oxalate ions, favoring its absorption and then its urinary excretion [95].

In terms of outcomes, a newer perspective on the effect of dietary calcium consumption on stone risk was provided in 1993. Dietary and urinary data from 45,619 years old male healthy individuals without stone events (Health Professionals Follow-up Study cohort) were analyzed, demonstrating that a low calcium diet was inversely associated with the risk for stone formation by 50% compared to a normal calcium consumption (797 ± 280 vs. 851 ± 307 mg of dietary calcium respectively) [96]. Although the former evidence was observational, a subsequent randomized clinical trial contrasted a balanced calcium consumption (1200 mg/day) coupled with low sodium and animal protein intake with a very low calcium diet (400 mg/day) in patients affected by recurrent calcium oxalate stones and hypercalciuria. The balanced calcium diet was confirmed to reduce both urinary oxalate excretion and the risk of new stone events after 5 years by approximately 50%. Moreover, as expected, patients in the low dietary calcium group had higher urinary oxalate excretion compared to subjects consuming a normal-calcium, low-salt, and low-animal-protein diet (60 μmol/day increase vs. 80 μmol/day decrease, respectively) [97]. Thus, a balanced calcium intake is now a mainstay of dietary recommendations for kidney stone formers, and its beneficial effect on kidney stone risk seems to be independent of its dietary origin [98].

On the contrary, the association between dietary calcium supplements and kidney stone risk is controversial. The clinical trial, “Women’s Health Initiative Calcium and vitamin D”, randomized 36,282 women in a post-menopausal state to receive either 500 mg of calcium carbonate and 200 UI of vitamin D3 two times per day or a placebo. The treatment group ingested an average of 2100 mg of dietary calcium per day and after 7 years of follow-up, these patients developed a 17% increase in the risk of stone formation compared to the placebo group [99,100]. Similar results were presented in an observational analysis of the Nurse’s Health Study I, showing a 20% increase in the risk of stone events for women assuming calcium supplements [101]. Of note, almost two-thirds of the patients in this study who took supplements between meals were associated with low oxalate consumption, possibly indicating that the timing of supplementation may have a role in this observation.

However, the following prospective studies (Nurse’s Health Study II, Health Professionals Follow Up Study) found no higher risk for stone events in patients assuming calcium supplementation [96,102,103].

To clarify the conflicting role of calcium supplementation, Domrongkitchaiporn et al. performed a cross-over study on 32 healthy subjects with a controlled diet randomly assigned to receive 1000 mg of calcium carbonate three times per day during meals or 3000 mg at bedtime. Urinary calcium excretion significantly increased in both bedtime and within-meal supplementation, without any difference between groups. However, when calcium supplements were taken during meals, urinary oxalate excretion was found to be decreased, which, in turn, was associated with lower calcium oxalate urinary supersaturation compared to bedtime consumption [104].

### 5.2. Vitamin D Supplementation

The effect of circulating calcitriol concentration on kidney stone risk is not a matter of debate. In fact, an analysis of the HPSF cohort by Taylor et al. demonstrated that after adjusting for potential confounders, patients who presented at least one episode of kidney stone events compared to controls had similar serum 25-hydroxycholecalciferol, whereas patients in the highest quartile for 1,25-dihydroxycholecalciferol and FGF-23 had significantly higher odds for stone events compared to the lowest quartile [56].

Although elevated calcitriol levels have been found to be deleterious to the risk of kidney stone formation, probably due to a direct increase in urinary calcium excretion, the association between serum 25-hydroxyvitamin D concentration, and thus of vitamin D administration, and incident kidney stone events is yet to be clarified.

Studies analyzing a causative role between serum 25-hydroxycholecalciferol and hypercalciuria in any phenotype of stone disease did not find any significant association. However, when only hypercalciuric kidney stone formers were taken into account, a correlation with vitamin D was found [55,105,106].

Epidemiological data from observational studies are conflictive: a cross-sectional analysis of the National Health Nutrition Examination Survey III including more than 16,000 men and women showed no association between 25-hydroxyvitamin D and self-reported history of nephrolithiasis [52].

However, when three bigger prospective cohorts were studied (Health Professionals Follow-up Study (HPFS), Nurses’ Health Studies (NHS) I, and NHS II), for a total of 193,551 participants, only the NHS II vitamin D supplementation group was linked to a slightly higher risk of kidney stone events (Hazard Ratio 1.18, *p* = 0.02). This may be due to the higher amount of prescribed vitamin D treatment in this cohort compared to the amounts used in the other studies [107].

Moreover, a recent meta-analysis investigated the role of circulating active and inactive vitamin D on the risk of incident nephrolithiasis. Hu et al. included 22 observational cohorts with 3,510 kidney stone formers and 19,718 healthy individuals. Patients were further categorized based on their stone phenotype (calcium or non-calcium based) and the presence or absence of hypercalciuria. Calcium stone formers had higher circulating calcitriol concentration compared to controls, whereas patients with hypercalciuria had both raised 1,25-dihydroxycholecalciferol and markedly increased 25-hydroxycholecalciferol. However, in normocalciuric stone formers, calcitriol was also found to be increased compared to controls. Based on these findings, the authors concluded that, while serum calcitriol concentration appear to be higher in all kidney stone phenotypes, 25-hydroxycholecalciferol is increased only in hypercalciuric stone formers [51].

Until now, evidence presented looked at the association between serum vitamin D concentration and the risk for kidney stone disease. However, several prospective studies also analyzed the consequence of direct vitamin D supplementation.

Kidney stone episodes were described as adverse events in two randomized placebo-controlled clinical trials on concomitant vitamin D administration and calcium supplementation [99,108].

In post-menopausal women affected by vitamin D insufficiency, the simultaneous administration of calcium citrate and vitamin D increased the risk of both hypercalcemia and hypercalciuria [109]. However, the administration of both calcium supplementation and vitamin D are expected to have completely different effects on urinary supersaturation. Dietary calcium intake and calcium supplementation during meals reduce urinary oxalate excretion, thus reducing calcium oxalate supersaturation, whereas calcium administration between meals and an excessive vitamin D supplementation might increase urinary calcium excretion without affecting oxaluria, thus increasing the risk for stone formation.

To answer this unresolved question, a meta-analysis from Malihi and colleagues [110] on 48 studies and more than 19,000 participants concluded that long-term vitamin D administration increased the risk for hypercalciuria up to 64% compared to placebo, although no difference in reported kidney stone events was found. Moreover, in a sub-group analysis, the risk for hypercalciuria was found to be independent of baseline circulating 25-hydroxyvitamin D concentration, vitamin D dosage, and concomitant calcium supplementation. However, there are only limited data on changes in urinary calcium excretion after initiating nutritional vitamin D supplementation, and the results were contradictory [111,112].

Nonetheless, these findings do not consider kidney stone phenotype and additional risk factors such as low urine volume, extremely acidic or alkaline urine pH, increased urinary excretions of oxalate and uric acid, or reduced urinary citrate concentration, probably explaining the observed heterogeneous results [14,16,18,113].

Recently, the Vitamin D Assessment Study, a randomized, placebo-controlled, double-blind clinical trial investigating the incidence of kidney stones and hypercalcemia in 5110 subjects randomized to receive a high monthly dose of vitamin D (100,000 IU/month) or placebo, concluded that after a median follow-up of 3.3 years, high-dose vitamin D administration does not increase the risk of stone formation and hypercalcemia in the general population [114]. Although this was a large study with an adequate follow-up time, it did not stratify patients based on stone phenotype and did not assess urinary and dietary risk factors for stone formation.

The genotype for some genes should also be considered as a possible contributor to the heterogeneous results of studies on vitamin D supplementation. Reports from Great Britain and Switzerland in the 1950s drew attention to an epidemy of hypercalcemia in infants associated with vitamin D-fortified milk [115]. An increased hypersensitivity to vitamin D was hypothesized, a hypothesis that has recently found support in the estimated high prevalence in the general population of homozygosity for mutated CYP24A1 appraised to be 420 to 1960 per 100,000 individuals [74]. Furthermore, as previously stated, heterozygous subjects might also be at risk of hypercalciuria and nephrolithiasis.

## 6. Conclusions

Kidney stone disease is a complex, multifactorial disease influenced by both genetic predisposition and environmental factors such as lifestyle and dietary habits. Different monogenic polymorphisms have been proposed as playing a causal role for calcium nephrolithiasis, including the VDR gene, CYP24A1, and SLC34A1; however, the complete pathogenetic pathway of hypercalciuria is yet to be determined. Balanced nutritional calcium consumption has been demonstrated to be protective from the risk of kidney stones by reducing intestinal oxalate availability and its urinary excretion. On the contrary, calcium supplementation, mostly if given between meals, might increase urinary calcium excretion without the beneficial effect on oxalate, thus increasing the risk for stone formation. Higher circulating active vitamin D has been reported in some kidney stone formers, whereas serum 25-hydroxycholecalciferol seems to be increased only in patients who already present with increased urinary calcium excretion. Literature regarding the role of nutritional vitamin D supplements in the risk for kidney stones is controversial, in part because the majority of these studies did not analyze additional risk factors and calcium consumption or supplementation. However, taken together, these observations might suggest that vitamin D administration worsens the risk for stone formation in patients predisposed to hypercalciuria. Additional prospective, diet-controlled clinical trials including 24,25-hydroxyvitamin D/25-hydroxyvitamin D ratio determination or genotyping for CYP24A1 should be performed to better investigate the role of mineral and bone derangements on the risk for kidney stones.

In practical terms, it is wise to evaluate the effect on calciuria a few weeks after vitamin D supplementation in each patient treated.

## Figures and Tables

**Table 1:** Foods with the highest calcium content.

Food Category	Calcium Content (mg per 100 g Serving)
Dairy	
Milk	276 mg
Cheese	From 138 to 333 mg
Kefir	247 mg
Buttermilk	222 mg
Yogurt	From 116 to 216 mg
Fish	
Sardines	286 mg
Salmon	From 179 to 212 mg
Vegetables	
Lambsquarters	362 mg
Nettles	334 mg
Amaranth	216 mg
Soybeans	175 mg
Spinach	154 mg
White beans	From 93 to 141 mg
Tofu	138 mg
Kale	94 mg
Almonds	93 mg
Broccoli	21 mg
Others	
Tahini	902 mg
Rice milk	221 mg

Data collected from the US Department for Agriculture (USDA 2002).
